# Can VHS Virus Bypass the Protective Immunity Induced by DNA Vaccination in Rainbow Trout?

**DOI:** 10.1371/journal.pone.0153306

**Published:** 2016-04-07

**Authors:** Dagoberto Sepúlveda, Niels Lorenzen

**Affiliations:** Department of Animal Science, Aarhus University, Aarhus, Denmark; INRA, FRANCE

## Abstract

DNA vaccines encoding viral glycoproteins have been very successful for induction of protective immunity against diseases caused by rhabdoviruses in cultured fish species. However, the vaccine concept is based on a single viral gene and since RNA viruses are known to possess high variability and adaptation capacity, this work aimed at evaluating whether viral haemorrhagic septicaemia virus (VHSV), an RNA virus and member of *Rhabdoviridae* family, was able to evade the protective immune response induced by the DNA vaccination of rainbow trout. The experiments comprised repeated passages of a highly pathogenic VHSV isolate in a fish cell line in the presence of neutralizing fish serum (*in vitro* approach), and in rainbow trout immunized with the VHS DNA vaccine (*in vivo* approach). For the *in vitro* approach, the virus collected from the last passage (passaged virus) was as sensitive as the parental virus to serum neutralization, suggesting that the passaging did not promote the selection of virus populations able to bypass the neutralization by serum antibodies. Also, in the *in vivo* approach, where virus was passaged several times in vaccinated fish, no increased virulence nor increased persistence in vaccinated fish was observed in comparison with the parental virus. However, some of the vaccinated fish did get infected and could transmit the infection to naïve cohabitant fish. The results demonstrated that the DNA vaccine induced a robust protection, but also that the immunity was non-sterile. It is consequently important not to consider vaccinated fish as virus free in veterinary terms.

## Introduction

Viral haemorrhagic septicaemia virus (VHSV) is a negative-sense, single-stranded RNA virus, which belongs to the *Novirhabdovirus* genus within the *Rhabdoviridae* family [1]. VHSV is the causative agent of the viral haemorrhagic septicaemia (VHS), a serious and economically important disease of farmed rainbow trout (*Oncorhynchus mykiss*) in Europe, causing high mortalities in all fish stages [2].

Currently, no commercial vaccine against VHS is available. Several vaccination strategies have been tested to control this disease, among them live attenuated vaccines, inactivated vaccines, and recombinant protein vaccines, but with limited efficacy or compromised safety aspects [3, 4]. By contrast, DNA vaccines have shown promising results by consistently protecting fish against VHS and related diseases [5, 6]. This led to the licensing and use of a DNA vaccine against infectious hematopoietic necrosis (IHN), caused by the related IHN virus, in Atlantic salmon in Canada since 2005, with no outbreaks reported since [7].

The traditional DNA vaccine against VHS consists of a plasmid designed for expression of the viral surface glycoprotein (G) in eukaryotic cells. The immune protection induced by this vaccine start as early as 4 days after intramuscular injection, when an early, non-specific, and short-lasting antiviral protection is triggered. This early protection has been related to interferon-associated mechanisms, which are characterized by the overexpression of multiples interferon-stimulated genes with antiviral functions such as Mx, Vig-1, Vig-2, and Vig-8 [8–18]. After 4–6 weeks depending on the water temperature, the temporary early protection is followed by the late, specific and long-lasting immunity, which includes the induction of both arms of adaptive immunity: the cell-mediated and the humoral responses [19–22].

Although the high efficacy of the DNA vaccine against VHSV has been consistent under experimental conditions, its protective effect might be threatened following repeated use under field conditions, due to the high variability of RNA viruses. Whether this variability might allow generation and selection of VHSV mutants, capable of evading the immunological protection induced by the DNA vaccine remains to be addressed.

Evidence of how the genetic variability of VHSV allows adaptation to selective conditions was shown when rainbow trout were injected with a plasmid encoding a neutralizing single chain antibody (scAb) against the G protein of VHSV. In this case, a neutralization escape mutant was isolated from the survivors of the infection [23]. Similarly, selective conditions provided by neutralizing monoclonal antibodies promoted the growth of neutralization-resistant virus variants in cell culture [24]. Similar observations have been made for IHNV [25].

While mutations in the G protein can affect the efficacy of the adaptive protection, mutations in e.g. the non-structural-protein (NV) may potentially affect the ability of the virus to bypass the innate protection induced by the DNA vaccine. This viral protein has been suggested to inhibit the apoptotic signal in virus infected cells at an early stage of virus infection, thus affecting the virulence of the VHSV [26].

The aim of this work was to determine whether VHSV within repetitive passages under the selective pressure of DNA vaccine-induced immunity, would be able to develop mutants that could escape from the early and late protective mechanisms induced by the vaccine, and from neutralizing serum antibodies induced in DNA-vaccinated rainbow trout. Knowledge of these aspects is essential for the design of robust and safe DNA vaccination strategies for protection of rainbow trout against VHS under field conditions.

## Materials and Methods

### Cells

The fish cell lines used in this study were EPC (epithelioma papulosum cyprini) [27] and BF2 (bluegill fry fibroblast) [28]. The cells were maintained in minimum essential media (MEM) supplemented with 10% fetal bovine serum (FBS), 100 U/ml of Penicillin and 100 μg/ml of Streptomycin. EPC and BF2 were grown for 24 h at 24°C and 21°C, respectively, and then maintained at 15°C.

### Virus

To propagate VHSV, BF2 cell cultures were inoculated with low multiplicity of infection of the virus and maintained at 15°C until a complete cytopathic effect (CPE) was observed. The supernatant was collected and centrifuged at 4500 x g for 15 min at 4°C to eliminate cellular debris. The virus was stored at -80°C. The titer was determined using the method of 50% tissue culture infective doses (TCID_50_) per ml, in BF2 cells [29].

### Animal experiments

All animal experiments were performed according to European and Danish rules for the use of experimental animals. The experiments were approved by Danish Animal Experiments Inspectorate under license No. 2014-15-0201-00379 carried by Niels Lorenzen. During periods with occurrence of clinical disease, the fish were monitored at least 3 times/day. Fish showing clinical diseases, such as exophthalmia, skin darkening, haemorrhages, or erratic swimming behavior, were terminated by extended immersion in anesthetic solution in order to reduce their suffering from disease to a minimum.

### Vaccination

For *in vivo* experiments, outbreed all female rainbow trout hatched and reared under pathogen-free laboratory conditions and with a weight of 2–8 g were used. For the vaccination, the fish were anesthetized in 0,01% benzocaine and injected intramuscularly (I.M.) in the left epaxial muscle below the dorsal fin with 25 μl of purified DNA plasmid in saline solution (0,9% NaCl), as described earlier [20]. This study included three vaccination conditions, the non-vaccinated fish, fish vaccinated with 0,1 μg, and fish vaccinated with 1,0 μg of the plasmid pcDNA3-vhsG. This vaccine consists of the glycoprotein gene of VHSV isolate DK3592b inserted downstream of a cytomegalovirus promoter in the eukaryotic expression vector pcDNA3 (Invitrogen). The plasmid construct was previously described [5, 6]. Non-vaccinated fish were used as controls. All fish were maintained in pathogen-free laboratory facilities in 120 l aerated aquaria supplied with recirculated water. One day before inoculation with virus (challenge), the fish were transferred to aerated aquaria of 8 l supplied with running tap water in a contained experimental facility. The average water temperature was 10°C throughout all vaccination and passaging/challenge experiments.

### Passaging of VHSV in vaccinated fish

Repeated passaging of VHSV was performed in fish vaccinated 1 week before inoculation with virus as well as in fish vaccinated 6 weeks before inoculation with virus. The fish were vaccinated with either 0,1 or 1,0 μg of the vaccine. Non-vaccinated fish were included as controls to confirm virulence the passaged virus.

In the first passage, each treatment group included two aquaria with 25 fish in each. The infection was carried out by immersion in 8 l water for 3 h in static water with a virus concentration of 1 x 10^5^ TCID_50_ ml^-1^ of the VHSV isolate DK3592b, hereafter called parental virus. After this, water flow was restored. Moribund fish, with clinical signs of VHS, were euthanized with an overdose of benzocaine and stored at −20°C until further analysis. At 21 days post infection, the surviving fish were euthanized with an overdose of benzocaine. The sampled moribund fish were dissected, and spleen, liver, heart, head kidney, and brain were collected and pooled per fish in MEM. Organs were homogenized in a TissueLyser (Qiagen) for 2 min at 20 Hz. The homogenate was centrifuged at 4500 x g for 15 min, and the supernatant was collected to be treated with gentamicin overnight at 4°C. After the antibiotic treatment, the virus content was titrated on BF2 cells and the samples stored at -80°C. These first-passage homogenates were used to infect new batches of fish vaccinated 1 or 6 weeks earlier (the second passage).

**Fig 1:**
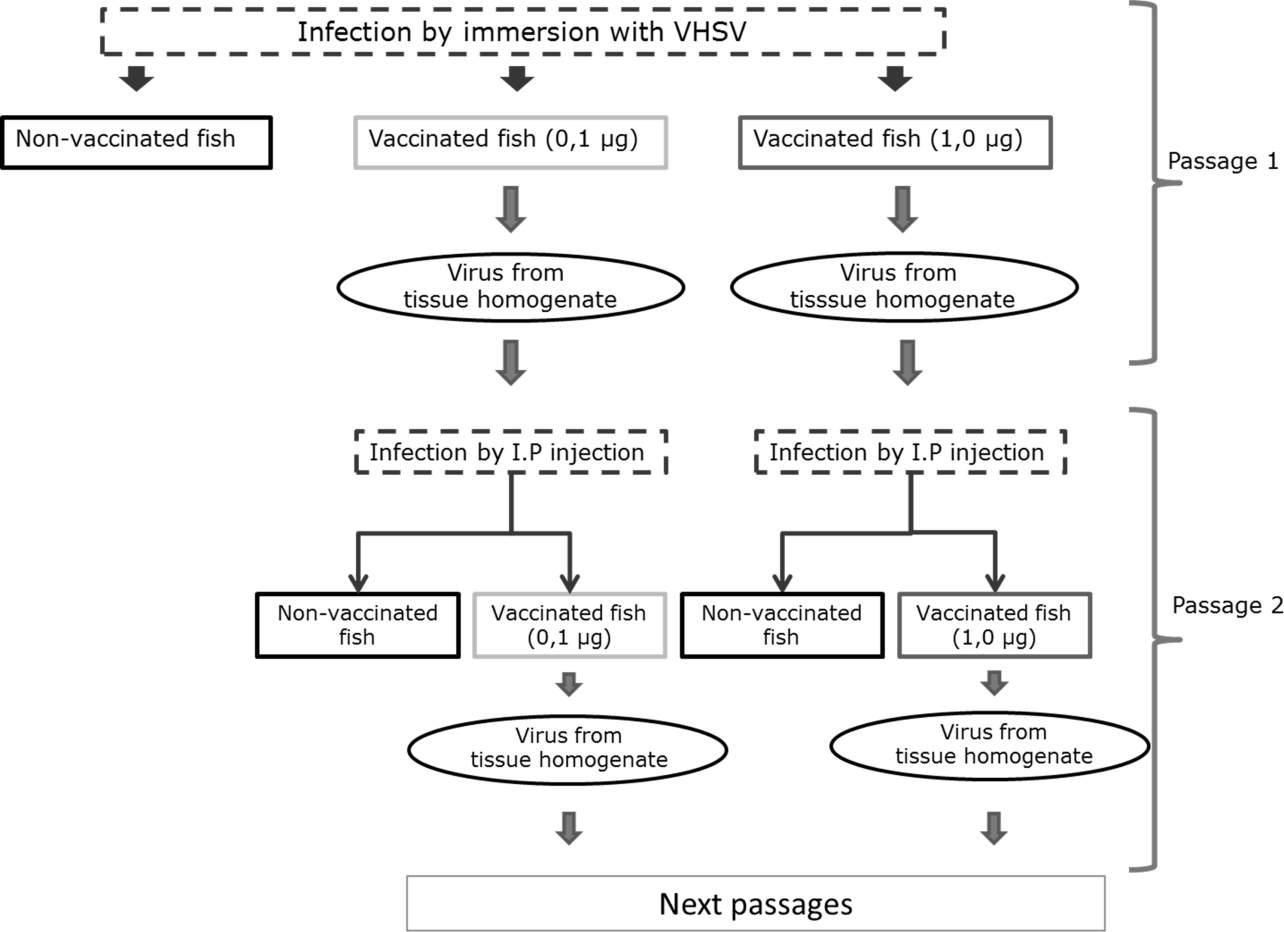
Schematic outline of the passaging of VHSV in vaccinated fish for the first two passages. Passage 3 and 4 followed the procedure of passage 2.

Due to the low amount of virus recovered from the first passage, the second passage was performed by I.P injection of 50 μl tissue homogenate supernatant into 2x10 fish. Subsequent passages were performed as the second passage with the exception that the tissue homogenates included organs from both survivors and moribund fish. The procedure for monitoring and sampling was performed as explained in the first passage (Fig 1).

### Examination for virus escaping from vaccine-induced immunity

A challenge trial with vaccinated fish was performed to compare the performance of the parental virus with the virus from the last passage in vaccinated fish, hereafter called passaged virus, under the selective pressure induced by DNA vaccination. To obtain sufficient virus for immersion challenge, the passaged virus, was passaged once in BF2 cells and titrated as outlined above.

The comparative analyses included accumulated mortality, the virus carrier state of the vaccinated fish, as well as the ability of the virus to spread to co-habitant naïve fish. The fish were vaccinated with 1,0 μg of the DNA vaccine and challenged at 1 or 6 weeks post-vaccination, using virus passaged at similar times post-vaccination. Fish challenged with the parental virus served as controls. As an example, when the fish were challenged at 1 week post-vaccination the passaged virus used was VHSV-1W0,1 and VHSV-1W1,0. With each virus, the infection was performed in duplicates with 20 fish in each aquarium. The infection was carried out by immersion in static water in the 8 l aquaria for 3 h with a final concentration of the virus at 1x10^4^ TCID_50_ ml^-1^. After this, the water flow was restored. Two weeks post-infection, 10 non-vaccinated (naïve) fish were transferred into each aquarium to cohabitate with the vaccinated fish. The naïve fish were tagged by cutting a part of the tail fin. After another 2 weeks, all surviving fish were euthanized with an overdose of anesthetics. The spleen, heart, liver, kidney and brain were collected and pooled from each fish individually. The tissues were homogenated and used for virological examination. The persistence in vaccinated fish and the spread of the virus to naïve fish were calculated as the percentages of virus-positive fish.

### Virological examination

Supernatants from tissue homogenates treated as described above were used to prepare 10-fold serial dilutions, which were inoculated onto BF2 monolayers with 60–80% confluency in 24-well tissue culture plates. The cultures were maintained at 15°C for 7 days when each well was examined. The wells with CPE were considered positive. The identity of the CPE-causing virus was confirmed by PCR as outlined below.

### Confirmation virus identity

An RT-PCR assay with primers specific for the VHSV nucleoprotein (N)-gene was used to confirm the identity of the passaged virus as well the virus re-isolated from sampled fish in the comparative challenge trial. Total RNA was isolated from 100 μl of each supernatant using the RNeasy mini kit (Qiagen). Subsequently, the cDNA was synthesized using the iScript kit (BioRad) following the manufacturer's recommendation. The qPCR reaction contained 1 μl of the cDNA, 7,5 μl of SuperMix, 0,03 μl of Rox, 0.3 μl of each primer VHSV-N-For 5’-AGG TCT CAG ATG TCA TCA AGG AG-3’ and VHSV-N-Rev 5’-CGG TGG AGC TCC TGA AGT T-3’, and 5.87 μl of free-nuclease water. PCR amplification program involved an initial step at 50°C for 2 min, and then a denaturation cycle 95°C for 2 min. These steps were followed by 40 cycles 95°C for 15 sec and 60°C for 1 min. Amplification was performed in MX Pro-Mx3005P thermocycler (Stratagene). Samples giving a signal within 25 cycles and an amplicon product with a melting temperature corresponding that obtained with the positive VHSV isolate DK3592b control, were considered as positive.

### *In vitro* passaging of VHSV in the presence of neutralizing trout serum

For the first passage, serial 10-fold dilutions of VHSV isolate DK3592b (30 μl) were mixed in wells of 96-well plates with 15 μl of a 1/40 dilution of the heat treated (30 min at 45°C) neutralizing trout serum obtained after repeated immunization with the DNA vaccine (pcDNA3-vhsG) [19]. Following incubation for 1 h at 15°C, 15 μl of a 1/40 dilution of normal trout serum was added per well as complement source [30]. After incubation overnight at 15°C, 10 μl of the treated virus was added to the wells of a 96-well plate with EPC cell culture, seeded the previous day, and incubated for 30 min at 15°C. Finally, to maintain the selective environment, 50 μl of MEM 5% FBS supplemented with a dilution of 1/640 of the immune serum and a dilution of 1/640 of the complement was added. Five replicate wells were used for each virus dilution. The cells were incubated at 15°C for 7 days. After that, we collected the supernatant from 3 wells given the lowest virus inoculum still causing CPE. Serial dilutions were made with each of the 3 collected supernatants. The dilutions were mixed with the neutralizing trout serum for a second passage, following the same procedure as for the first passage, except that only 3 replicate cell culture wells were inoculated with diluted antiserum/virus mixtures. This passaging under selective (antiserum) pressure was repeated 11 times.

### Evaluation of *in vitro* passaged virus

The susceptibility of the *in vitro* passaged virus and the parental virus to the neutralizing effect of the immune serum was compared using the plaque neutralization assay (PNT), in which the titer of the serum is defined as the reciprocal value of the highest serum dilution reducing the number of plaques by 50% compared to a normal trout serum control [30]. To evaluate the presence of mutations, the full-length glycoprotein gene of each passaged virus was amplified by PCR and sequenced. Total RNA isolation and cDNA synthesis was performed following the same procedures outlined above. The PCR amplification was performed with primers flanking the G gene using the high fidelity DNA polymerase Herculase II Fusion (Agilent Technology) according to the procedure recommended by the manufacturer. The annealing step in the PCR was performed at 57°C, using the forward primer 5´ TAC AAT CGT GCC GTC GAA G 3´ and the reverse primer 5´ AGG TCA CAG TTG AGG TAG TTG 3´.

### Statistical analysis

Each replicate in both the virulence and the persistence analyses was considered as an individual sample to evaluate intergroup variability. The statistical analysis for difference between treatments was performed by the Kruskal-Wallis test (R Studio, version 0.98.501). Differences were considered significant at p<0.05.

## Results

### *In vivo* approach: Evaluation of the ability of passaged VHSV to evade the early and late protection induced by the DNA vaccine

#### Passaging in vaccinated rainbow trout

To test the ability of the virus to bypass the early protection induced by the DNA vaccine, we passaged VHSV in rainbow trout vaccinated 1 week before inoculation with virus. When the fish used for passaging had been vaccinated with a dose of 0,1 μg of the DNA vaccine, we were able to isolate virus from dead and vaccinated surviving fish in each of the successive 5 passages. When fish given 1,0 μg of the DNA vaccine were used, we were able to isolate virus only until the 2^nd^ passage. This might suggest that the protection induced by 1,0 μg of the DNA vaccine was able to clear the virus in the immunized fish more efficiently than in fish vaccinated with 0,1 μg DNA, although the latter dose was still highly protective (data not shown).

To test the ability of the virus to bypass the late protection induced by the DNA vaccine, the passaging was performed in rainbow trout vaccinated 6 weeks before inoculation. In this setup, VHSV was re-isolated from dead and vaccinated survivor fish in all 4 passages using both vaccine doses (Table 1).

**Table 1:** Passaged virus.

Virus Label	Passages	Status of fish used for passaging	
		Time post vaccination***	Vaccine dose
VHSV-1W*0,1**	5	1 week	0,1 μg
VHSV-1W1,0	2	1 week	1,0 μg
VHSV-6W0,1	4	6 weeks	0,1 μg
VHSV-6W1,0	4	6 weeks	1,0 μg

* Refers to the number of weeks between the vaccination and challenge

** Refers to vaccine dose (μg)

*** Time post-vaccination when the challenge was performed

#### Comparison of passaged and parental virus

After successive passages, the virus obtained from the last passage under selective conditions (passaged virus), was amplified by 1 passage on BF2 cells and later compared with the parental VHSV isolate.

The comparison for the *in vivo* approach included three factors; mortalities induced in vaccinated and non-vaccinated fish, the capacity of the virus to infect and persist in vaccinated fish, and the ability of the virus to spread from vaccinated carriers to cohabitant naïve fish.

**Fig 2:**
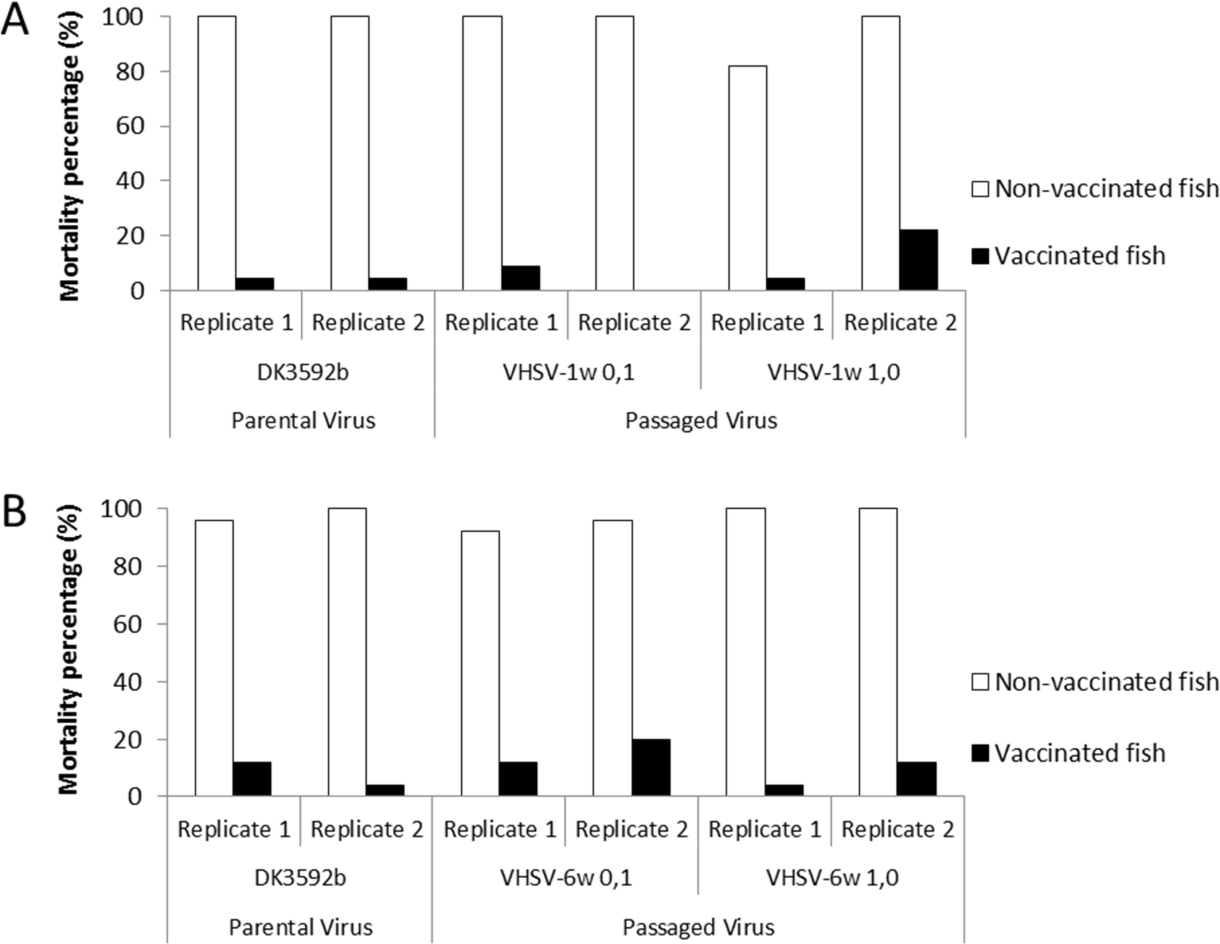
Comparison of the mortality of rainbow trout challenged with the parental virus and the passaged virus. The challenges were performed at 1 week post-vaccination **(A)** and at 6 weeks post-vaccination **(B)**. White bars correspond to non-vaccinated fish and black bars correspond to fish vaccinated with the DNA vaccine. The mortality values of the vaccinated fish infected with the passaged and the parental virus at either 1 or 6 weeks post-vaccination were not statistically significant different. The mortality values of the non-vaccinated and the vaccinated fish challenged at either 1 or 6 weeks post-vaccination were statistically significant different (p<0,005).

The mortality rates caused by the passaged virus and the parental virus in vaccinated fish were both low, ranging between 0–12%. Only one of the duplicate aquaria with fish inoculated with the VHSV-1W1,0, and one of the duplicates inoculated with the VHSV-6W0,1, showed higher mortalities (about 20%) than the aquaria inoculated with the parental virus (Fig 2). Since this increased virulence was not observed in both duplicates, it was considered to be due to an intergroup variability rather than increased virulence of the passaged virus. Accordingly, no statistical difference in the virulence of passaged and parental virus could be detected (p<0,05). It cannot be fully excluded that the slightly higher mortality in two of the individual aquaria inoculated with the passaged virus could be due to escape mutants occurring during the final challenge test. However, sequencing of the G-gene did not reveal any changes (not shown). In non-vaccinated fish, all batches of passaged virus induced high mortality rates, indicating that the passaging had not affected the virulence.

The evaluation of the ability of the virus to infect vaccinated fish showed that both the parental and passaged virus were able to infect and persist in some of the vaccinated fish for at least 4 weeks post-challenge (Table 2). The highest frequencies of carriers in individual aquaria were obtained with the parental virus, with 36% and 15,8% of virus-positive fish, when the evaluation was performed at 1 and 6 weeks post-vaccination, respectively. Lower values were found among the fish inoculated with passaged virus, where the highest carrier frequencies reached 13.6% and 10.5%, respectively. Therefore, the passaging had not increased the ability of the passaged virus to persist in vaccinated fish. The observed carrier frequencies were accordingly found not to be significantly difference (p<0.05).

**Table 2:** Comparison of the carrier status of vaccinated rainbow trout challenged with the parental virus or with the virus passaged in vaccinated fish.

Challenge time	Virus	Replicate Aquaria N°	Vaccinated fish positive in virological examination (%)	Cohabitant naïve fish positive in virological examination **
1 wpv*	Parental virus	1	36,4	++++
		2	5,6	-
	VHSV-1w 0,1	1	5,6	-
		2	13,6	-
	VHSV-1w 1,0	1	0	-
		2	5,0	++++
**6 wpv**	Parental virus	1	0	-
		2	15,8	+++
	VHSV-6w 0,1	1	10,5	+++
		2	5	+++
	VHSV-6w 1,0	1	0	-
		2	10,0	-

* wpv: Weeks post vaccination

** Relation frequencies of virus positive fish: “++++” All fish were positives, “+++” more than 50% of the fish were positive, and “-” none of the fish were positives.

The analysis of whether vaccinated carriers infected with either the passaged or the parental virus were capable of transmitting the infection to naïve fish showed that even though only a few fish could be detected as carriers, transmission did take place for the passaged as well as for the parental virus, in at least in one of the replicates (Table 2). The setup did not allow detailed risk analyses and there was not a direct relation between the number of fish carrying the virus and the isolation of virus from naïve cohabitant fish within the individual aquaria. While VHSV-1w0,1 was re-isolated from 13,6% of the vaccinated fish in one replicate aquarium, no infection among the naïve co-habitants was observed. In another aquariumVHSV-1w1,0 was re-isolated from 5% of the vaccinated fish and from all the cohabitant naïve fish. The high intergroup variability could be due to e.g. individual variability in virus secretion by the carriers. Also, it has to be taken into account that the evaluation was performed 2 weeks after cohabitation and 4 weeks after infection, and it may be anticipated that the frequency of vaccinated fish carrying the virus at the time of introduction of cohabitants could have been higher than when analysed 2 weeks later. Further experiments including higher numbers of fish and time-course studies are required to solve this, but the results clearly demonstrate that vaccinated fish exposed to VHSV must be considered to be potential carriers and transmitters of the infection.

### *In vitro* approach: Evaluation of the ability of passaged VHSV to escape from neutralization by serum from fish immunized with the DNA vaccine

After 11 successive passage in cell culture in the presence of neutralizing trout serum, we compared the ability of the passaged virus and the parental virus to escape from the neutralization by a trout immune serum. As shown in Table 3, no significant difference in susceptibility to neutralization was found between the passaged virus and the parental virus.

**Table 3:** Neutralizing titers of trout immune serum for *in vitro* passaged and parental virus.

	Parental Virus DK3592b		Passaged Virus1		Passaged Virus2		Passaged Virus3	
	IS	CS	IS	CS	IS	CS	IS	CS
50% PNT titer *	5120	<40**	20480	<40	5120	<40	10240	<40

IS = Immune serum collected from rainbow trout immunized with the DNA vaccine; CS = Control serum collected from non-vaccinated rainbow trout

* Neutralization titer was expressed as the reciprocal of the dilution of the antibody reagent reducing the plaque number by 50%

**The value <40 was considered as no virus neutralization.

The glycoprotein gene of the 3 preparations of passaged virus and the parental virus was sequenced and compared to determine the presence of mutations. The 4 sequences had 100% nucleotide identity (not shown).

## Discussion

This work focused on analyzing whether the fish rhabdovirus VHSV was able to mutate and escape from the protective mechanisms induced by the VHS DNA vaccine. Such mutants would represent a potential risk of reemergence of the disease in vaccinated fish populations, reducing the applied potential of the DNA vaccine. To our knowledge, this important aspect remains to be addressed for the otherwise extensively analyzed and highly protective fish DNA vaccines. Our results suggest that the immune response triggered by the vaccine is rather robust and not easily bypassed by the virus.

The high genetic variability of RNA viruses is due to the high replication rates, large population size, and high mutation rates, which together generate a diverse spectrum of virus variants in every replication cycle and result in a population of one dominant virus variant along with multiple low-frequency virus variants. This phenomenon is known as the quasispecies theory and allows the virus to adapt rapidly to new environments, and to new hosts [31]. When RNA viruses are exposed to selective exogenous conditions, the frequency of some virus variants in the population could change, favoring those with a certain advantage to replicate in the new condition. The fact that VHSV has been found in a wide range of host fish species, along with the ability of the virus to easily escape from neutralization by monoclonal antibodies, suggests a high adaptation capacity [2, 23, 24, 32]. We, therefore, questioned whether the virus would also be able to adapt to DNA-vaccinated fish.

The experimental design included two approaches. First, an *in vivo* approach was used to evaluate the ability of VHSV to evade the early and late protection induced by the DNA vaccine in rainbow trout fingerlings, associated with innate and adaptive immunity, respectively [22, 33, 34]. Secondly, an *in vitro* approach was applied, to evaluate the ability of the virus to evade the neutralizing effect of serum from rainbow trout immunized with the DNA vaccine.

In the *in vivo* passaging, the initial inoculation of the fish was done by immersion. i.e. the natural route of infection. But from the second passage, the inoculation was performed by I.P injection due to the low amounts of virus recovered from each passage, and to avoid propagation of the virus in cell culture, which could compromise the selection procedure. In order to obtain enough virus to perform the infection in the final evaluation trial by immersion, one cell culture passage was required. Although this was a compromise, the risk of this significantly changing the virus population after several passages under selective conditions *in vivo* was considered to be small. Sequence analysis by next generation sequencing would be required to confirm this, but was beyond the scope of the present study.

The analysis of the *in vivo* approach took into account that an escape mutant could have diverse strategies to bypass the immune protection induced by the DNA vaccine. Among these strategies, escape mutants could have increased virulence in vaccinated fish, causing higher mortality rates as a result of a more efficient viral replication. Alternatively, decreased virulence might reduce clearance, thereby allowing the virus to persist in the host and spread to cohabitant fish. However, the *in vivo* approach evaluation showed no advantage of any of the batches of passaged virus in comparison with the parental virus, neither considering virulence, persistence in vaccinated fish, nor the capacity of vaccinated fish carriers to transmit the infection to naïve cohabitant fish.

According to Read et al. 2015, infected vaccinated chickens were associated with a higher risk of spread of highly virulent virus than the infected non-vaccinated chickens. The rationale behind this theory was that non-vaccinated chickens would die out rapidly and thereby allow the infection to be kept under control, while the infected vaccinated chicken would survive the infection and allow the virus to persist, replicate and spread in the host population for an extended period. Furthermore, the persistence in vaccinated chickens could promote the selection of hyperpathogenic virus strains that could cause a more severe disease [35].

While this scenario might be true for some highly lethal viruses, our results suggest that it does not count for VHSV infections in rainbow trout. Although the virus causes high mortality, some individuals often survive and clearance from such fish is slower compared to DNA vaccinated individuals [36]. Therefore, since we were unable to isolate escape mutants after repeated passaging in vaccinated fish, repeated stocking with vaccinated fish seems to be a viable strategy for reducing the prevalence of VHSV in endemic zones. The animal experiments presented here were all conducted at an average water temperature of 10°C. Host pathogen interactions in fish are often highly dependent water temperature and we cannot exclude that different results would be obtained at higher or lower temperatures. However, at 10°C rainbow trout is highly susceptible to VHS and outbreaks in the field often occurs around this water temperature [37], which was therefore considered to be appropriate for the present study. Inoculation of vaccinated fish was performed at two times post-vaccination, at 1 and 6 weeks corresponding to 70 and at 420 degree-days (temperature in °C times days post vaccination), respectively. Previous work has demonstrated that at 70 and 420 degree-days, protective mechanisms induced by VHS DNA vaccine base on innate, and a combination of innate and adaptive elements, respectively [14, 22, 38], and as discussed further below, our results suggest that VHSV cannot easily bypass these protective mechanisms.

The *in vitro* aim was look at the adaptive humoral immune response to DNA vaccination alone and evaluate whether serial passages of VHSV in the presence of serum from rainbow trout immunized with the DNA vaccine, would favour propagation of neutralization escape mutants. The trout immune serum used here was obtained from a hyperimmunized fish [6].

Previous works showed that viruses like VHSV and IHNV were able to generate escape mutants resistant to neutralization by monoclonal antibodies after a few passages in cell culture [24, 25]. Another setup showed that it was possible to isolate an escape mutant from rainbow trout injected with a plasmid encoding a neutralizing recombinant single chain antibody (scAb) against the glycoprotein of VHSV [23].

However, after 11 passages, there was no evidence of mutants escaping the neutralizing effect of the trout immune serum. This was confirmed by the fact that the passaged and the parental virus had a 100% identical glycoprotein gene sequence. In contrast to this, mutants escaping neutralization by mouse monoclonal antibodies carried one or few amino acid substitutions in the translated G gene [24]. The explanation for why the apparently rather adaptable virus could not escape from the neutralization by our fish immune serum might be due to different neutralization mechanisms by mouse monoclonal antibodies (IgG1) and by fish polyclonal IgM antibodies in serum. Neutralizing monoclonal antibodies bind a single epitope and thereby most likely interfere with the process of infection, e.g. by preventing recognition of the receptors on the cell membrane or generating aggregates incapable of infecting host cells [39]. In contrast, to produce a mutant escaping the neutralizing serum antibodies the virus may have to mutate at multiple sites, assuming that vaccination induces a polyclonal neutralizing response. Furthermore, fish IgM neutralizing activity depends on the presence of complement, implying that the neutralization mechanism is not just a steric blocking of the viral infectivity, but a more complex mechanism potentially involving different sites of the protein, which could be more difficult for VHSV to bypass without affecting the functional biology of the virus particle [40, 41]. In summary, the results from the *in vitro* analysis suggested that VHSV cannot easily escape even from the neutralizing serum antibodies, which is only one mechanism of the adaptive responses induced by DNA vaccination. However, while a single vaccination did not induce an antibody response in all individuals, particularly at lower temperatures [22], our failure to isolate escape mutants *in vivo* suggested that the broad nature of the immune response triggered by the vaccine, involving a range of both innate and adaptive mechanisms, made escape mutation incompatible with maintaining the viability and the infectious capacity of the virus.

Our setup only included the most stringent selective condition by challenging vaccinated fish with virus carrying a G gene identical to the vaccine gene. It can therefore not be excluded that viral escape from DNA vaccine induced immunity might arise under less selective conditions, such as when the vaccine G gene is heterologous to that of the infecting virus, resulting in a less complete protection [20]. The safest strategy would thus be to sequence the prevalent VHSV variants in the fish population to be vaccinated and then perform the vaccination with a homologous or at least genetically closely related vaccine gene.

In conclusion, our results suggest a low probability of occurrence of escape mutants under optimal DNA vaccination conditions. In applied terms, this represents an additional advantage of this rather efficient vaccine against VHSV, making it a safe prophylactic tool. However, we also observed that some of the vaccinated fish can get subclinically infected and that the infection can be transmitted to naïve cohabitants if these are stocked with the vaccinated fish shortly after their exposure to the virus. Vaccinated fish from endemically infected zones should therefore be considered to be potential carriers in terms of trade regulations.
